# Effectiveness of vaginal probiotics *Lactobacillus crispatus* chen-01 in women with high-risk HPV infection: a prospective controlled pilot study

**DOI:** 10.18632/aging.206032

**Published:** 2024-07-25

**Authors:** Yujuan Liu, Xiumiao Zhao, Fei Wu, Jie Chen, Juanzhen Luo, Chunling Wu, Tingtao Chen

**Affiliations:** 1 Department of Gynaecology, The Affiliated Hospital of Jiangxi University of Traditional Chinese Medicine, Nanchang, Jiangxi 330006, People’s Republic of China; 2 Department of Gynaecology, The First Hospital of Nanchang, Nanchang, Jiangxi 330006, People’s Republic of China; 3 Queen Mary School, Nanchang University, Nanchang 330031, People’s Republic of China; 4 Department of Obstetrics and Gynecology, The Second Affiliated Hospital of Nanchang University, Nanchang, Jiangxi 330006, People’s Republic of China; 5 Department of Gynaecology, Jingdezhen Maternal and Child Health Care Hospital, Jingdezhen, Jiangxi 333000, People’s Republic of China; 6 School of Pharmacy, Jiangxi Medical College, Nanchang University, Nanchang, People’s Republic of China; 7 National Engineering Research Center for Bioengineering Drugs and The Technologies, Institution of Translational Medicine, Jiangxi Medical College, Nanchang University, Nanchang, People’s Republic of China

**Keywords:** HR-HPV, vaginal microbiota, cervical cancer, probiotic transplantation, *Lactobacillus crispatus* chen-01

## Abstract

Female genital tract infection with high-risk human papilloma virus (HR-HPV) has the risk of developing into cervical cancer, and there is still a lack of effective therapeutic strategies. Probiotic intervention is considered as a potential intervention for HR-HPV, while exploration into living probiotic preparations for specific diseases remains limited and insufficient. This prospective controlled pilot study was conducted to observe the effect of intravaginal transplantation of a vaginal isolated natural probiotic strain, *Lactobacillus crispatus* chen-01, on the clearance of high-risk HPV infection. 100 women with high-risk HPV infection were enrolled and randomly divided into placebo group and probiotic treatment group, which received intravaginal transplantation of *L. crispatus* chen-01. Cervical exfoliated cells were collected 6 months later for detecting DNA load, typing of HPV, and cytological analysis. Our results showed that vaginal transplantation with *L. crispatus* chen-01 significantly reduced viral load of HPV, ameliorated HPV clearance rate, and improved vaginal inflammation state without causing obvious adverse reactions. Analysis of 16S rRNA sequencing revealed that *L. crispatus* chen-01 could effectively reconstitute the vaginal microbiota in women with high-risk HPV, which might be one of the underlying mechanisms of the beneficial effect of *L. crispatus* chen-01 transplantation. Our results suggested that vaginal transplantation of *L. crispatus* chen-01 might be a promising treatment for patients with high-risk HPV infection.

## INTRODUCTION

Human papillomavirus (HPV) infection is one of the most common sexually transmitted diseases of the female lower genital tract worldwide. The prevalence of female genital HPV infection ranges from 12% to 49.5% showing regional variation [1, 2], meanwhile, around 80% of women have the chance to be exposed to HPV during lifetime [3]. Genital HPVs can be subdivided into high- and low-risk types, and persistent high-risk HPV infection gives rise to cervical intraepithelial neoplasia (CIN) or even cervical cancer. It has been reported that almost all invasive cervical cancer biopsies contain intact HPV-DNA [4], thus, eradication of HPV infection is the key to preventing CIN and cervical cancer. Currently, inoculations of HPV vaccine are the main way to prevent HPV infection. However, even the most popular nine-valent vaccine does not fully cover all high-risk types. Therefore, the lack of treatments for those who have been infected with HPV promote the urgency to develop safe and effective anti-HPV methods in the prevention and treatment of cervical cancer.

The vagina is an outside-communicating channel for sexual intercourse and menstruation, which connects upwards to the cervix, and downwards to the external environment. It is colonized by a plethora of microorganisms, including aerobic bacteria, anaerobic bacteria, known as the commensal microbiota, which interact with each other to keep the balance of vaginal microbiota and a healthy state [5]. *Lactobacillus* is the dominant bacterial species in the reproductive tract of healthy women, which produces lactic acid, hydrogen peroxide, and bacteriocins to maintain an acidic environment and prevent pathogen growth, adheres on the epithelium, repels other bacteria adhesion, and regulates immune and inflammatory response, enhancing the resistance of vagina to diseases [6]. However, vaginal microbiota is susceptible to various influence factors, such as menstrual cycle, sexual activity, and douching [7]. Once the balance is disrupted, it leads to various vaginal inflammatory diseases, such as bacterial vaginosis (BV), vulvovaginal candidiasis (VVC), and sexually transmitted diseases including HPV infection [8, 9].

Recently, increased evidence has suggested that the abnormal vaginal microbiota plays a role in the acquisition and persistence of HPV infection and elevates the risk of cervical cancer [10]. High-throughput sequencing of vaginal secretions revealed significant differences of vaginal microbiota between HPV-negative and HPV-positive women, which manifests as a highly diverse vaginal microbiota with a decrease of *Lactobacillus* spp. and increase of *Gardnerella vaginalis* [11]. Normally, *Lactobacillus* prevents the colonization of species related to bacterial vaginosis by maintaining low pH and producing bacteriocins, which is important for cervical epithelial barrier function of inhibiting HPV from entering basal keratinocytes [12]. When dysbiosis of vaginal microbiota happens, the number of *Lactobacilli* reduces, and *Gardnerella* as well as anaerobic bacteria colonize massively and release enzymes and metabolites, which may damage the cervical epithelial barrier and provide opportunities for HPV infection and invasion [11, 13]. HPV infection is closely related to the dysbiosis of local vaginal microecology, especially the reduction of *Lactobacilli* [14]. Thus, restoring vaginal microecological balance with *Lactobacillus* supplementation is expected to be a potential and promising therapeutic strategy for HPV infection.

Probiotics are defined as “living microorganisms that can confer health benefits to the host when applied in sufficient quantities” [15], which exert beneficial effects through various mechanisms, including modulating pH, decreasing the colonization and invasion of other pathogenic organisms, as well as modifying the host immune responses [16]. At present, probiotics and their products have been widely used in gastrointestinal disorders, allergic diseases, diabetes, cardiovascular diseases and cancers [17, 18]. In the female reproductive tract, the most common probiotics are *Lactobacillus* spp., including *L. crispatus, L. gasseri, L. iners,* and *L. jensenii* [18]. However, there are few commercial vaginal probiotics currently available in the market. In China, the only available vaginal probiotic is the *Living Preparation of Lactobacillus*, which is composed of non-human origin *L. delbrueckii* with low bacterial content. Meanwhile, the application of probiotic products in gynecological diseases is limited, and prescription drugs mainly target the treatment of bacterial vaginosis. Therefore, the potential use of probiotics in gynecological diseases including interventions on HR-HPV infection needs to be further explored.

Considering the scant awareness on the clinical use of probiotics in gynecological diseases, few research on the local transplantation of *Lactobacilli* for the treatment of gynecological diseases has been reported, potentially hindered by the difficulty in the large sample recruitment and the ethical limitations on the clinical research. Although studies in the animal stage have been proved effective [19], the compositional difference between vaginal microbiota in humans and animals, as well as its correlated mechanism greatly limited the further research. Moreover, since the traditional oral administration route may undergo the first elimination of the liver, and how it adjusts the vaginal microecological balance is still unclear, the mode of administration remains to be considered. Encouragingly, with the development of fecal microbiota transplantation in full swing, local transplantation of *Lactobacilli* has aroused broad concern. Benefiting from the application of high-throughput sequencing, roles of single-strain probiotics in the treatment of gynecological diseases have also been continuously explored. In this study, we take the lead to testify the role of *L. crispatus* chen-01, a natural probiotic strain isolated from the healthy human in the intervention of HR-HPV infection. 100 subjects with high-risk HPV infection were recruited, and the clinical safety and efficacy of intravaginal administration of *L. crispatus* chen-01 were evaluated. High-throughput sequencing was used to analyze the diversity and alteration of vaginal microbial composition. Our study explored the correlation between cervical HR-HPV infection and vaginal microecology, as well as the role of probiotic intervention in the treatment of HR-HPV, which provides a novel strategy for the treatment and management of cervical HR-HPV infection, and a promising concept for the prevention and treatment of cervical intraepithelial neoplasia and cervical cancer.

## RESULTS

### Basic characteristics of the participants

**Figure 1:**
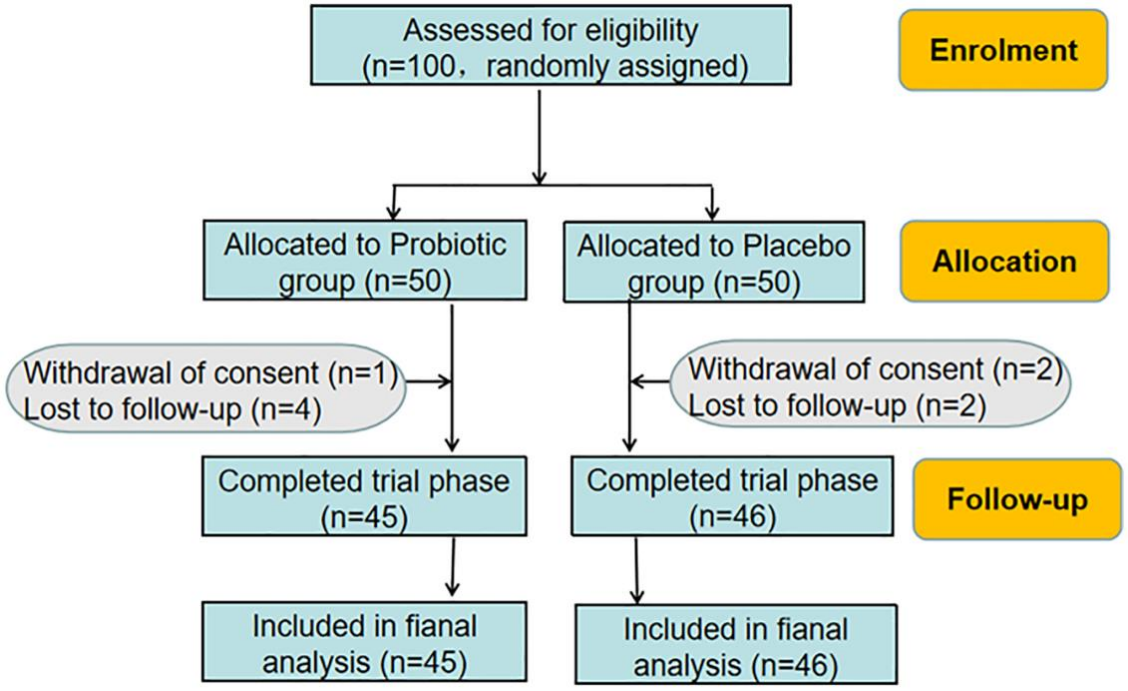
Flowchart of the trial.

According to inclusion and exclusion criteria, 100 individuals were recruited in the trial and were randomly divided into Probiotic group and Placebo group, each with 50 cases respectively. Further, baseline characteristics of the patients in two groups were evaluated. The baseline information (Table 1) showed that there was no significant difference between the two groups in terms of age (40.34 ± 1.77 vs. 38.88±1.64), the history of pregnancy (2.70 ± 0.22 vs. 2.50 ± 0.21) and childbirth (1.50± 0.12 vs. 1.42 ± 0.13), smoking, contraceptive methods, age of initial sexual behavior (19.92 ± 0.33 vs. 19.6 ± 0.36) and number of sexual partners. There was also no statistical difference in the initial HPV viral loads (263.60 ± 25.15 vs. 237.4 ± 25.17), mental stress (6.06 ± 0.15 vs. 5.94 ± 0.14), symptoms prevalence, physical exercise and the family history of cervical cancer. During the study, 1 case withdrew from the consent and 4 cases were lost to follow up in Probiotic group while 2 cases withdrew from the consent and 2 cases were lost to follow up in Placebo group. Finally, 45 cases in Probiotic group and 46 cases in Placebo group completed the trial phase (Figure 1).

**Table 1:** Patients’ baseline characteristics (Mean ± SD).

**Variables**	**Probiotic group (*n* = 50)**	**Placebo group (*n* = 50)**	***p*-value**
**Age**	40.34 ± 1.77	38.88 ± 1.64	0.55
**The number of pregnancy**	2.70 ± 0.22	2.50 ± 0.21	0.51
**The number of delivery**	1.50 ± 0.12	1.42 ± 0.13	0.65
The way of delivery			0.79
Vaginal delivery	37	34	
Caesarean section	6	8	
No delivery	7	8	
**Use of contraceptive methods**			0.85
Condom USE	16 (32.00%)	14 (28.00%)	
IUD	19 (38.00%)	20 (40.00%)	
Hormonal contraception	10 (20.00%)	12 (24.00%)	
**Age of initial sexual activity**	19.92 ± 0.33	19.6 ± 0.36	0.51
**Smoking**	6 (12.00%)	4 (8.00%)	0.51
**Number of lifetime partners**			0.60
1–2	10	8	
≥3	40	42	
**Level of stress on a scale of 10**	6.06 ± 0.15	5.94 ± 0.14	0.56
**Symptoms prevalence** (vaginal itching and burning, dyspareunia and dysuria)	8 (16.00%)	6 (12.00%)	0.56
**Physical exercise**			0.57
Never	10	13	
Once to twice a week	35	30	
>Twice a week	5	7	
**Initial viral load** (RLU/PC)	263.60 ± 25.15	237.4 ± 25.17	0.46
**With cervical cancer in the family**	3 (6.00%)	4 (8.00%)	0.70

### Vaginal probiotics *L. crispatus* chen-01 effectively improve the clinical outcome of HPV infection

First of all, the effect of the use of *L. crispatus* chen-01 for 5 months on HPV viral load in the 6th month was evaluated, and the results were shown in Table 2. Before probiotic treatment, the initial HPV viral loads of patients in Probiotic group and Placebo group were 267.70 ± 27.68 and 245.10 ± 26.43, respectively, and there was no significant difference at the beginning (*P* = 0.56). After treating with *L. crispatus* chen-01 in Probiotic group for 5 months, the viral load of patients was 113.10 ± 21.69, which was significantly lower than that before treatment (*P* < 0.001), as well as the Placebo group in 6th month (202.90 ± 27.10, *P* < 0.05). Although viral load of both two groups shows significantly reduce (*P* < 0.001), there is significant difference between two groups in the 6th month (*P* = 0.01).

**Table 2:** HPV viral load before/after treatment in two groups (Mean ± SD).

**Groups**	**Before**	**After**	***P*-value**
Probiotic group (*n* = 45)	267.70 ± 27.68	113.10 ± 21.69	<0.001
Placebo group (*n* = 46)	245.10 ± 26.43	202.90 ± 27.10	<0.001
*P*-value	0.56	0.01	

Furthermore, the HPV clearance rate in the follow-up 6th month was compared in Table 3. In the 6th month, 21 patients in Probiotic group turned negative, and 5 cases of multiple infection were partially negative which was considered effective; while 19 patients remained positive, with a total effective HPV clearance rate of 57.78%. In the Placebo group, 17 patients turned negative, 4 patients were effective and 25 patients were positive, with a total effective HPV clearance rate of 45.65%. The total effective HPV clearance rate in probiotics group was 12.13% higher than that of the Placebo group (*P* > 0.05).

**Table 3:** HPV clearance rate before/after treatment in two groups (Mean ± SD).

**Groups**	**Cure**	**Effective**	**Ineffective**	**Clearance rate (%)**
Probiotic group (*n* = 45)	21	5	19	57.78
Placebo group (*n* = 46)	17	4	25	45.65
*P*-value				0.25

Then cytological and inflammatory changes of the two groups were compared in Table 4. The analysis of cervical exfoliative cytology showed that in the 6th month, the cytological improvement rate in the Placebo group was 34.62%, and the inflammation improvement rate was 27.27%. However, as for Probiotic group, cytological improvement rate was 82.14% and vaginitis improvement rate was 77.78%, both of which were significantly higher than that of Placebo group (*P* < 0.05). In addition, there were no adverse events reported in this study.

**Table 4:** Cytological and inflammatory changes before/after treatment in two groups (Mean ± SD).

**Groups**	**Before**		**After**		**Cytological improvement rate (%)**	**Vaginitis improvement rate (%)**
	**Cytological abnormality**	**Inflammation**	**Cytological abnormality**	**Inflammation**		
Probiotic group	28	9	5	2	82.14	77.78
Placebo group	26	11	17	8	34.62	27.27
*P*-value					<0.01	0.02

### Intravaginal treatment with *L. crispatus* chen-01 in HR-HPV patients decreases the diversity of vaginal microbiota

**Figure 2:**
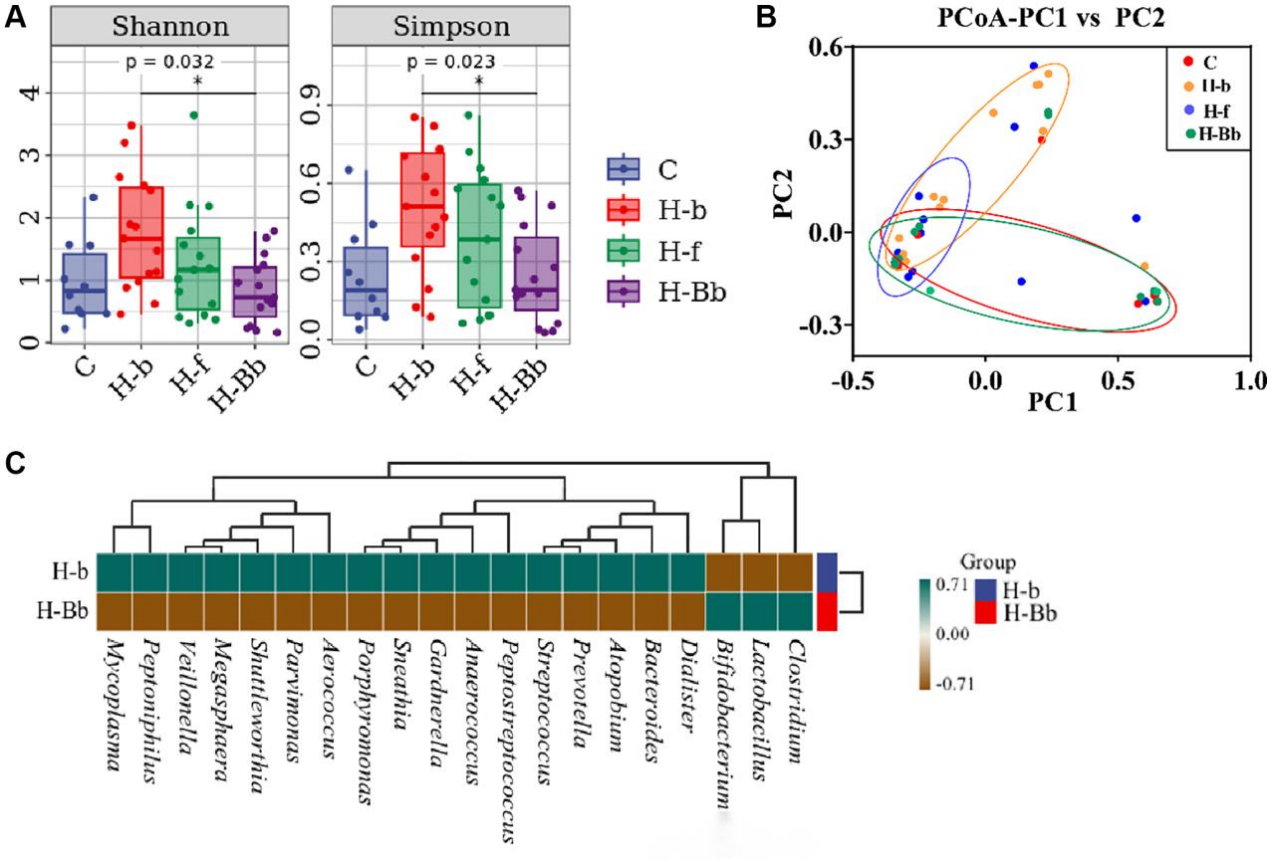
**Effects of intravaginal transplantation of *L. crispatus* chen-01 on the diversity and abundance of vaginal microbiota.** (**A**) Comparison of Shannon and Simpson in the groups of C, H-b, H-f and H-Bb. (**B**) PCoA analysis among the groups of C, H-b, H-f and H-Bb. (**C**) Heatmap of species composition between before (H-b) and after (H-Bb) probiotic treatment. The abbreviated descriptions are as follows: C: Healthy people without HPV infection; H-b: Patients of HR HPV before enrollment; H-f: Patients of HR-HPV after treatment with placebo; H-Bb: Patients of HR HPV after treatment with probiotic. ^*^P < 0.05; ^**^P < 0.01.

In order to further explore the influence of probiotic intervention on the vaginal microbial composition, Shannon and Simpson indexes were used to assess the alpha diversity of microbial communities. As shown in Figure 2A, Shannon (0.84 ± 0.14 vs. 1.76 ± 0.24, *P* = 0.032) and Simpson (0.25 ± 0.05 vs. 0.50 ± 0.06, *P* = 0.023) indexes were significantly reduced in HR-HPV patients after 5 months’ probiotic treatment (H-Bb group) compared with those of HR-HPV patients before enrollment (H-b group). Furthermore, the alpha diversity of microbial communities in group H-Bb with probiotic treatment is similar to that of the healthy people without HR-HPV infection (C group). As for β-diversity, we used principal coordinate analysis (PCoA) to display discrepancy among the four groups (Figure 2B). As shown in Figure 2B, most of the points in the group H-b before probiotic intervention are scattered away from group C, which means that there is a significant deviation of the microbial compositions of HPV infection individuals compared with those of the healthy population. However, after intravaginal treatment with *L. crispatus* chen-01 for 5 months, the points of probiotic treatment group (H-Bb group) clustered closer to those of the normal healthy population (group C), indicating a more similar species diversity of vaginal microbiota between the two samples.

In order to further compare the differences of species composition among samples and evaluate the distribution trend of species abundance in each sample, the heat map was also used to analyze the species composition. As shown in Figure 2C, the level of *Lactobacilli* increased obviously in patients in H-Bb group after 5 months probiotic treatment compared with that before intervention in H-b group (0.56 ± 0.10 vs. 0.96 ± 0.01, *P* < 0.01), which means the normal vaginal microbiota is effectively established.

### Intravaginal treatment with *L. crispatus* chen-01 significantly alters vaginal microbial composition at phylum and genus levels

**Figure 3:**
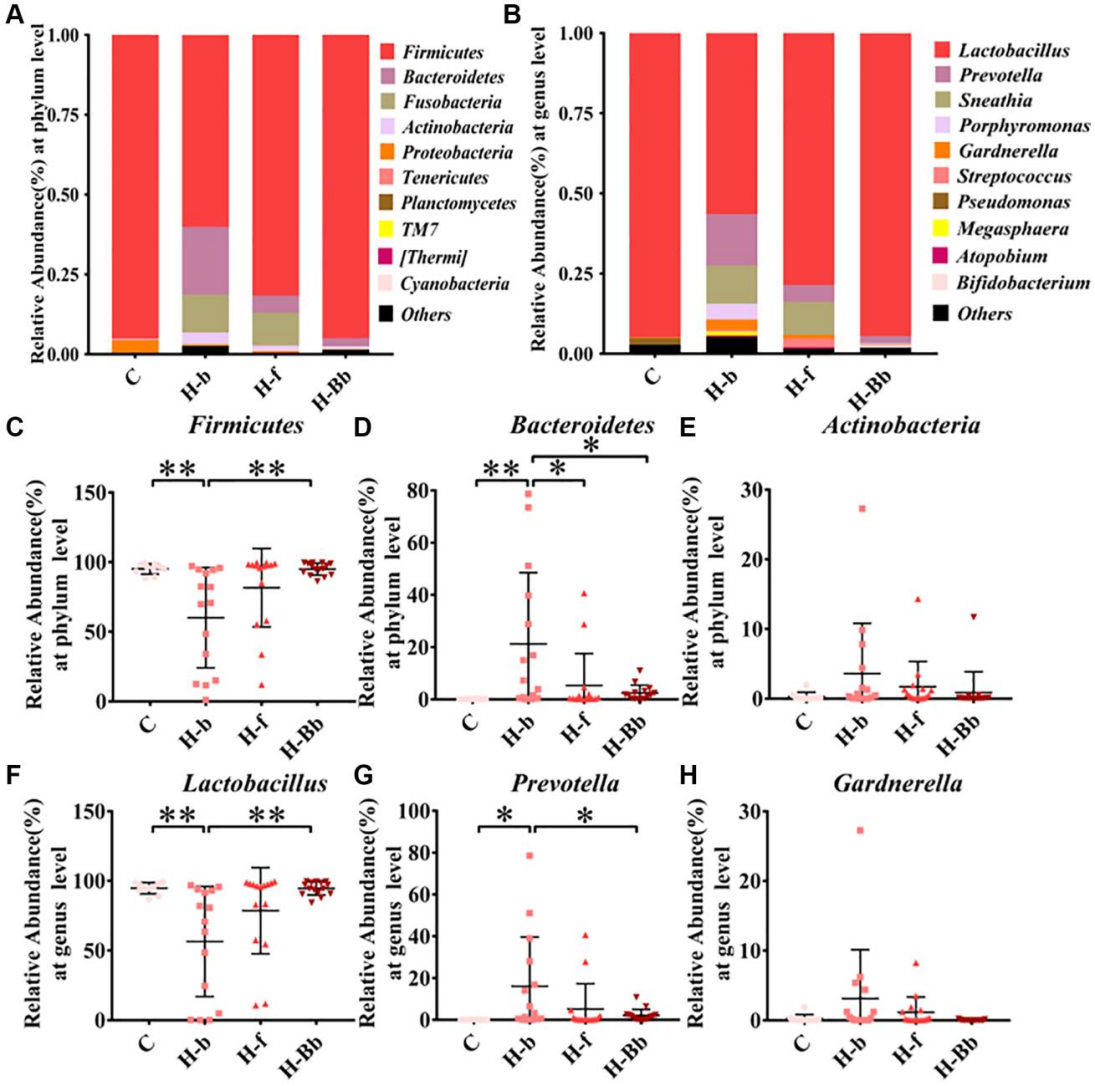
**Effects of intravaginal transplantation of *L. crispatus* chen-01 on vaginal microecological composition.** (**A**) Comparison of vaginal microecological composition at the phylum level in the groups of C, H-b, H-f and H-Bb. (**B**) Comparison of vaginal microecological composition at the genus level in the groups of C, H-b, H-f and H-Bb. (**C**–**E**) Comparison of phylum composition of *Firmicutes*, *Bacteroidetes* and *Actinobacteria* in the groups of C, H-b, H-f and H-Bb. (**F**–**H**) Comparison of genus composition of *Lactobacillus, Prevotella and Gardnerlla* in the groups of C, H-b, H-f and H-Bb. C: Healthy people without HPV infection; H-b: Patients of HR HPV before enrollment; H-f: Patients of HR-HPV after treatment with placebo; H-Bb: Patients of HR HPV after treatment with probiotic. ^*^P < 0.05; ^**^P < 0.01.

To explore the differences in vaginal microbiota pre and post *L. crispatus* chen-01 treatment, we further analyzed the community composition of vaginal microbiota in different groups at the phylum and genus level. As shown in Figure 3A, the vaginal microbiota of the normal healthy population (group C) was dominated by *Firmicutes* accounting for 95.13% (95.13 ± 1.19) at the phylum level, while the remaining phyla accounting for less than 5%. However, *Firmicutes, Bacteroidetes, Fusobacteria* and *Actinobacteria* were the top four phyla in HR-HPV patients comprising more than 99% of the total sequences, indicating an increased bacterial diversity after HPV infection. In Figure 3C–3E, when compared with healthy state (group C), the richness of *Firmicutes* in vaginal microbiota of HR-HPV patients reduced (60.10 ± 9.31 to 95.13 ± 1.19, *P <* 0.01), while after treatment with *L. crispatus* chen-01, the abundance of *Firmicutes* significantly increased (94.96 ± 1.11 to 60.1 ± 9.31, *P* < 0.05). However, there is an opposite trend observed between *Bacteroidetes* and *Actinobacteria*. In addition, we found that HR-HPV patients with *L. crispatus* chen-01 treatment restore vaginal microbiota to normal state after treatment.

At the genus level, Figure 3B showed that *Lactobacillus* was the dominant vaginal bacteria accounted for 94.74% (94.74 ± 1.28) in the healthy population (group C), while *Prevotella*, *Sneathia* and *Gardnerella* only account for small proportion. Compared with group C, the proportion of *Lactobacillus* in HR-HPV patients decreased, while the remaining bacteria increased. When treating with *L. crispatus* chen-01 in HR-HPV patients (group H-Bb), the abundance of *Lactobacillus* restored to normal level. In Figure 3F–3H, the abundance of *Lactobacillus* in HR-HPV patients (H-b group) reduced (56.45 ± 10.21 to 94.74 ± 1.28, *P <* 0.01) compared with samples from healthy states (group C), while *Prevotella* and *Gardnerella* showed the opposite trend, which could be reversed by the supplementation of *L. crispatus* chen-01, manifesting as a significant increase in *Lactobacillus* abundance in the H-Bb group (94.50 ± 1.22 to 56.45 ± 10.21, *P* < 0.01) and a downward trend of *Prevotella and Gardnerlla* (H-f and H-Bb groups). These results showed a decreased vaginal microbiota abundance post treatment in HR-HPV patients, which means local application of *L. crispatus* chen-01 significantly improved vaginal microbial composition and was conducive to restore normal vaginal microbiota.

## DISCUSSION

Cervical cancer (CC) is the fourth most common malignancy in women worldwide [20]. It is well established that a persistent infection with high-risk HPV (HR-HPV) causes nearly all cases of invasive cervical cancer [21]. Therefore, eradicating HPV is expected to be an effective way to prevent cervical cancer. Recently, emerging literatures have highlighted the significance of cervicovaginal microbiota in the HR-HPV susceptibility and clearance [22], which indicates regulating cervicovaginal microbiota might be an effective treatment strategy for HP-HPV infection.

Since HR-HPV infection with high-grade cervical intraepithelial neoplasia, cervical cancer or tumors in the genital tract require clinical intervention such as surgical or medical treatment, in order to testify the effectiveness of involving vaginal microbiota in the treatment of HPV infection, we recruited high-risk HPV infection cases mostly without cervical lesions or diagnosed as low-grade cervical intraepithelial neoplasia and administered a natural probiotic stain isolated from the vagina in the healthy individuals—*L. crispatus* chen-01 locally to observe the alterations of clinical parameters and vaginal microbiota. In the clinical practice, we observed the ameliorative effect of intravaginal transplantation of *L. crispatus* chen-01 on patients with HR-HPV infection. Our results showed a significant reduction in HPV viral load and a 1.3-fold increase of HPV clearance rate in patients who received *L. crispatus* transplantation compared with the control group. Although both of the two groups showed reduction of viral load, there are significant difference between the two groups in the follow-up 6th month, which means that the application of probiotics facilitated the clearance of HPV in addition to the natural clearance. Our result is consistent with Pierro’s study that oral administration of *L. crispatus* could change the vaginal CST status in patients and improve the clearance rate of HPV [23]. Similarly, Dellino et al. also showed that total HPV clearance increase from 9.3% to 15.3% in the patients taking long term oral *L.* M247 compared with those who didn’t receive following interventions [24]. Vaginal microbiota transplantation could reconstitute healthy vaginal microbiota, which had a certain effect on bacterial vaginosis [25]. Previous research also indicated that the long-term vaginal administration of *Lactobacilli* in HPV-infected patients with bacterial vaginitis or vaginitis help to eliminate not only vaginitis but also the viral infection [26]. Here, our result further confirmed that the use of vaginal probiotics *L. crispatus* chen-01 could effectively improve vaginitis and cervical exfoliative cytology without causing severe adverse reactions.

In order to analyze the possible mechanism of HPV clearance by *L. crispatus*, high-throughput sequencing was used to analyze the diversity of cervicovaginal microbiota and the changes of vaginal microbial composition pre and post treatment of *L. crispatus*. Recent cross-sectional studies have revealed a higher diversity of cervicovaginal microbiota in HPV-positive women compared with that of HPV-negative individuals [27, 28]. Our analysis further indicated an increase of *Prevotella* and *Gardnerella*, but a decrease of *Lactobacillus* in high-risk HPV cases, which is consistent with the research performed by Dong et al. that the overgrowth of *Prevotella* may participate in the occurrence of persistent HR-HPV infection-related cervical lesions [29]. Encouragingly, by giving *Lactobacillus* locally, this trend was expected to be reversed. A healthy vaginal microbiota is mainly composed of 1 - 2 *Lactobacillus* species, showing low species diversity [5]. Our results showed that probiotic treatment significantly reduced the diversity of vaginal microbiota both at the phylum and genus levels, similar to those of the healthy female.

In the normal female reproductive tract, vaginal microbiota is dominated by *Lactobacillus* spp., accounting for 70–95% [5]. *Lactobacillus*-dominated microbiota has long been considered as the hallmark of health in the female reproductive tract [30]. Among this, *L. crispatus* can inhibit the growth of various vaginal pathogens and fungi, improve antibiotic sensitivity, and reduce the risk of sexually transmitted diseases [31]. These properties of *L. crispatus* may be related to its capacity to adhere on the host cells to prevent pathogen colonization, produce bactericidal substances (lactic acid, hydrogen peroxide), niche occupancy, and immune regulation [6]. Studies have shown that *L. crispatus* can promote the proliferation of vaginal epithelial cells and the healing of damaged epithelium, thus maintaining the integrity of the vaginal epithelial barrier [32]. As an immunomodulator, *L. crispatus* also participates in the immunomodulation of the cervicovaginal microenvironment, which plays an important and positive role in preventing pathogen infection and even inhibiting the carcinogenesis of cervical cells [33, 34]. Although the barrier function of *Lactobacilli* can inhibit the entry of HPV into basal keratinocytes, not all *Lactobacillus*-dominant cervicovaginal microbiota is protective. Lactic acid has D- and L-isomer, while D-lactate is more effective in maintaining the vaginal homeostasis [35]. For example, D-isomers can inhibit the decomposition of extracellular matrix by down-regulating matrix metalloproteinase 8 (MMP-8), which prevents the entry of HPV into the basal keratinocytes by altering cervical integrity [36]. Moreover, it can inhibit histidine deacetylase and activate gene transcription of local innate immune molecules in vagina [36].

**Figure 4:**
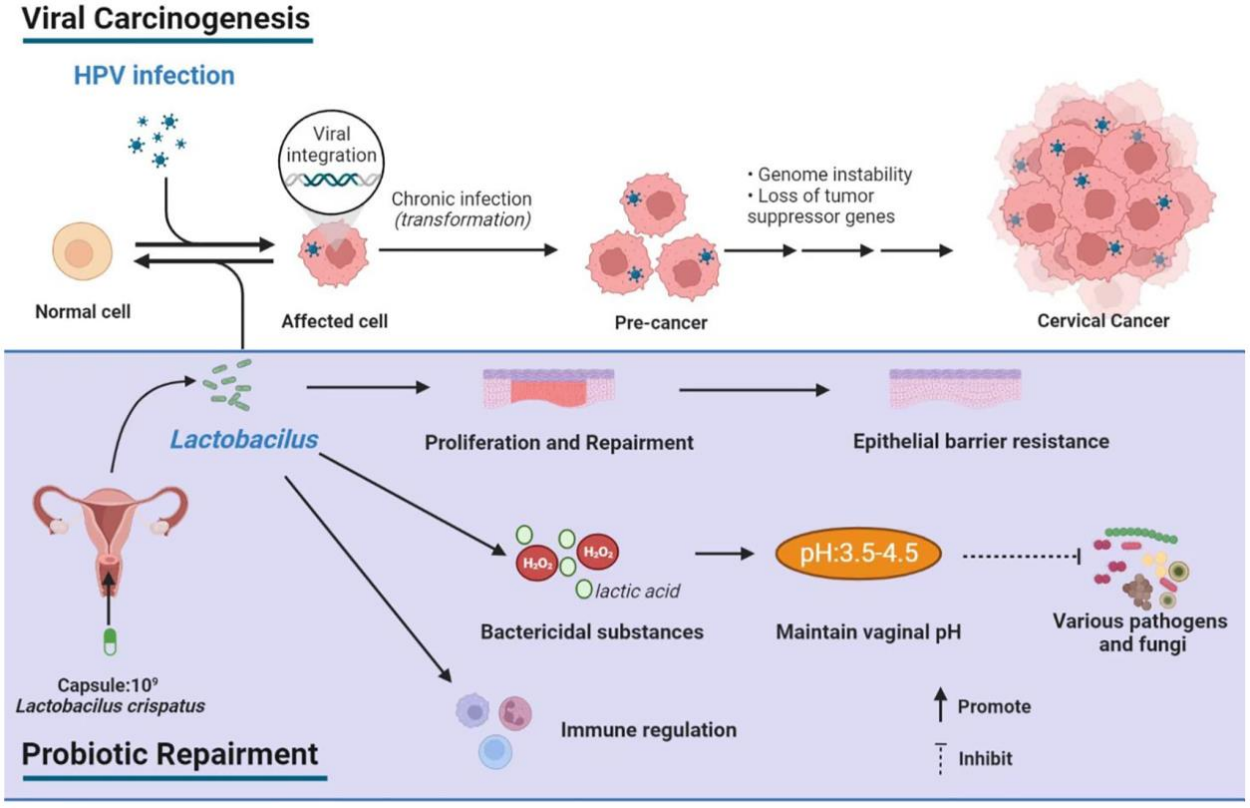
Schematic depiction of viral carcinogenesis and probiotic repairment.

*L. crispatus, L. jensenii* and *L. gasseri* produce both D- and L-lactate, but *L. iners* and a variety of anaerobes can only synthesize L-lactate [37]. Given the quantity and stability of *L. crispatus* in vaginal cervicovaginal microbiota, as well as its strong ability to produce D-lactate and H_2_O_2_, a natural isolated *L. crispatus* strain was selected as the objects of our study. The anti-HPV mechanism of *Lactobacillus* is shown in Figure 4, indicating *L. crispatus* has a promising protective effect on the female reproductive tract, whose dysbiosis strongly correlates with the development of various genital tract inflammation and even HPV infection.

Through high-throughput sequencing analysis, lower levels of *Lactobacilli* and higher levels of *Prevotella* and *Gardnerella* has been found in patients with HR-HPV compared to that of the healthy people. After *L. crispatus* treatment, the abundance of *Lactobacilli* increased, while *Prevotella* and *Gardnerella* decreased, which tend to be similar to those of the normal healthy population. However, the mechanism by which *Prevotella* and *Gardnerella* promotes HR-HPV infection remains unclear.

*Prevotella* is a Gram-negative anaerobe, which is one of the dominant bacteria in human vaginal microbiota and is an opportunistic pathogen, causing diseases such as bacterial vaginosis [38]. *Prevotella* can produce sialidase (SNA), a group of mucin-degrading enzymes, which disrupts the integrity of mucosa as well as the epithelium to help the entry of HPV to basal keratinocytes [22, 39]. Based on transkingdom network analysis and the result of the co-culture with a cervical cancer cell line, *Prevotella* is found to upregulate LAMP3, a key tumor driver, which plays a crucial role in cervical cancer by driving expression of multiple antiviral genes under anaerobic conditions [40]. In addition, overgrowth of *Prevotella* in the vagina may influence the occurrence of persistent HR-HPV infection-related cervical lesions through host NF-κB and C-myc signaling [29].

*Gardnerella* is the most common microorganism identified from the vaginal samples in women with BV. It is one of the most common bacteria in the vaginal microecology of patients with HPV infection and cervical cancer [41]. It can not only form dense biofilms to adhere tightly on the surface of vaginal epithelial cells, release cytolysin and activate inflammatory responses, but also release metabolites such as producing sialidase (SNA), hemolysin to destroy vaginal epithelial cells, damage the mucosal barrier, and promoting the adhesion and invasion of pathogens, such as HPV [42, 43]. Thus, increased *Gardnerella* is regarded as a key co-factor of HPV infection to cervical carcinoma or precancerous lesions.

In this study, we evaluated the efficacy of intravaginal administration of *L. crispatus* in the treatment of patients with HR-HPV. The results showed that *L. crispatus* could significantly reduce the HPV viral load, improve the HPV clearance rate, improve conditions of vaginitis with no severe adverse reactions. Furthermore, *L. crispatus* chen-01 could effectively reconstitute the vaginal microbiota in women with HR-HPV. These results suggest that intravaginal administration of *L. crispatus* is a potential effective and specific treatment for patients already infected with HR-HPV. However, the underlying mechanisms of the improved clearance rate can be sophisticated, which can be achieved by the bacterial interactions, competitive adhesion or the secretion of bacterial products, etc. Therefore, exploration into the potential mechanism underlying the improved clearance rate is a further task in our following research. This treatment is simple, feasible with no obvious side effects, which has the value of clinical promotion. However, there are still limitations of this study such as the relatively short observational time, thus the long-term therapeutic effects of *L. crispatus* transplantation remains to be further evaluated. Moreover, difficulty in the recruitment of large samples make it harder to control the potential heterogeneity of the participants, thus hindering the current clinical research. This requires a more comprehensive study to make the further confirmation.

## MATERIALS AND METHODS

### Study design and participants enrollment

This study was a double-center, double-blind, placebo-controlled trial. Female patients with cervical HR-HPV infection were recruited from gynecological outpatients or inpatients unit of the First Hospital of Nanchang and Jingdezhen Maternal and Child Health Care Hospital from July 1, 2021 to June 30, 2022. Patients who met the following criteria were included in the study: voluntary to be enrolled in the study; female, aged between 18 and 65 years old, having history of sexual life; without vaginal douching, medicine use and sexual life 3 days before enrolling in this study; having no immune diseases and metabolic diseases such as diabetes. Meanwhile, patients with high-grade cervical intraepithelial neoplasia, cervical cancer or genital tract tumors, history of hysterectomy, history of allergy to drugs or probiotics were excluded. Participants who had been taking contraceptives, antibiotics, immunosuppressants or other drugs in the last 3 months were also excluded. All subjects underwent clinical evaluation on the necessity of the medical intervention, and have performed colposcopy, and cervical biopsy when necessary. Regular follow-up visits were performed according to the clinical guidelines. Basic characteristics of the participants such as age, history of pregnancy and childbirth, smoking, contraceptive methods, age of initial sexual behavior and number of sexual partners were collected (Table 1). All patients who participated in this study have signed informed consent. This study has been approved by the Medical Ethics Committee of the hospital and registered on the official website of the Chinese Clinical Trial Registry (registration number: ChiCTR2100046239).

### Patients grouping and implementation of clinical trial protocol

100 HR-HPV patients were recruited in the program based on inclusion and exclusion criteria. According to the ratio of 1:1 with random number table method, eligible patients were randomly divided into two groups: the Probiotic group and the Placebo group. The probiotic preparation was a mixture of *L. crispatus* chen-01 (No. CGMCC 23396) and sweet-potato-flour, with 1 × 10^9^ colony-forming units (CFU) per capsule of live bacteria [44]. The placebo contained only the same grams of sweet-potato-flour. Patients in Probiotic group were given probiotic preparation capsule locally in the vagina as the following described: after cleaning the vulva, use a gloved finger to place the capsule into the deep vagina, one capsule each time and once every night. Use for 14 days continuously in the first three months. In the next fourth and fifth month, use it once every three days and five times a month. The usage in Placebo group was the same as that of the Probiotic group. Do not douche vagina during medication and avoid menstruation.

### Collection of samples, detection of HPV DNA and thinprep cytologic test (TCT)

Methods of collecting samples are as follows: firstly, the cervix was exposed with a vaginal speculum; then, vaginal secretions were wiped out with a sterile cotton ball, then a special brush was rotated clockwise 3 times and was removed and placed into the special preservation solution. One part of the sample was used for HPV DNA detection, and the other part was used to test the cytological classification by TCT. The hybrid capture II (HC II) was used to detect the HR-HPV types: 16, 18, 31, 33, 35, 39, 45, 51, 52, 56, 58, 59 and 68, and HPV-DNA ≥1 pg/ml was considered as positive criteria. Real-time fluorescence quantitative PCR was used to detect HPV type. Criteria of cure was defined as all HPV subtypes turning negative. Effective was defined as one or more HPV subtypes turning negative. Ineffective was defined as HPV subtype increased or no HPV subtype turning negative. HPV clearance rate (%) = cure rate (%) + effective rate (%). Inflammation state was assessed by the TCT examination results, which diagnose the inflammation state of high-risk HPV infection cases and categorize it into three levels—mild, moderate and severe. For TCT detection, the sample was rinsed to obtain cells, which were dispersed and filtered by automatic cell detector, and then subjected to microscopic detection and analysis.

### Sample collection, DNA extraction and high-throughput sequencing

Samples of vaginal secretions were collected on the 6th month after enrollment and 3 to 7 days after menstruation. Subjects were in a bladder lithotomy position, and the cervix was exposed by a professional gynecologist with a disposable vaginal speculum (the vaginal speculum was not coated with iodophor and other disinfectants). A sterile dry cotton swab was used to take a proper amount of vaginal secretions from the posterior fornix of the vagina and 1/3 of the lateral wall of the vagina, which were put into a sterilized tube, then sealed, marked and placed in a refrigerator at −80°C for high-throughput sequencing of 16S rRNA. Methods were provided by the technicians at Personal Biotechnology, Co., Ltd. (Shanghai, China). The total bacterial DNA of the sample was extracted, primers (F: AYTGGGYDTAAAGNG, R: TACNVGGGTATC TAATCC) were used to amplify the hypervariable V4 region of the 16S rRNA gene [45]. The PCR-amplified products were double-ended sequenced using the Illumina MiSeq platform (San Diego, CA, USA). Divisive Amplicon Denoising Algorithm 2 (DADA2) was used to obtain signature sequences of ASV/OTU, followed by processing using Quantitative Insights into Microbial Ecology (QIIME). The taxonomic classification was performed using the Greengenes database v13.8 and diversity analysis was performed by α-diversity, β-diversity, and species differences. Finally, 10 vaginal secretion samples were selected from healthy people without HPV infection (group C), and 45 vaginal secretion samples were randomly selected from HR-HPV patients before treatment (H-b group), after probiotic intervention (H-Bb group) and placebo treatment (H-f group), with 15 cases in each group. A total of 55 samples were used for high-throughput sequencing. The RNA-Seq data have been submitted to the NCBI Sequence Read Archive (SRA, http://www.ncbi.nlm.nih.gov/sra/) with SAR accession number PRJNA915656.

### Statistical analysis

All data generated in this study are expressed as mean ± standard deviation (SD), and analyzed or charted by GraphPad Prism (v8.0) and SPSS 22.0 (IBM Corp., Armonk, NY, USA). Qualitative data are expressed as rates. Paired and unpaired *t*-test was used for quantitative data and Fisher’s exact test or chi-square test for qualitative data to determine the significance between two groups of data. *P* < 0.05 is considered statistically significant.

### Data availability statement

The data that support the findings of this study are openly available in NCBI Sequence Read Archive (SRA, http://www.ncbi.nlm.nih.gov/sra/) with SAR accession number PRJNA915656.
